# Impact of climate variability on the transmission risk of malaria in northern Côte d'Ivoire

**DOI:** 10.1371/journal.pone.0182304

**Published:** 2018-06-13

**Authors:** Richard K. M’Bra, Brama Kone, Dramane P. Soro, Raymond T. A. S. N’krumah, Nagnin Soro, Jacques A. Ndione, Ibrahima Sy, Pietro Ceccato, Kristie L. Ebi, Jürg Utzinger, Christian Schindler, Guéladio Cissé

**Affiliations:** 1 Unité de Formation et de Recherche Sciences de la Terre et des Ressources Minières, Université Félix Houphouët Boigny, Abidjan, Côte d’Ivoire; 2 Centre Suisse de Recherches Scientifiques en Côte d’Ivoire, Abidjan, Côte d’Ivoire; 3 Swiss Tropical and Public Health Institute, Basel, Switzerland; 4 University of Basel, Basel, Switzerland; 5 Institut de Gestion Agropastorale, Université Péléforo Gon Coulibaly, Korhogo, Côte d’Ivoire; 6 Unité de Formation et de Recherche des Sciences Médicales, Université Félix Houphouët-Boigny, Abidjan, Côte d’Ivoire; 7 Centre de Suivi Ecologique, Dakar, Senegal; 8 International Research Institute for Climate and Society, Columbia University, New York, New York, United States of America; 9 Department of Global Health School of Public Health University of Washington, Seattle, Washington, United States of America; Institut Pasteur, FRANCE

## Abstract

Since the 1970s, the northern part of Côte d'Ivoire has experienced considerable fluctuation in its meteorology including a general decrease of rainfall and increase of temperature from 1970 to 2000, a slight increase of rainfall since 2000, a severe drought in 2004–2005 and flooding in 2006–2007. Such changing climate patterns might affect the transmission of malaria. The purpose of this study was to analyze climate and environmental parameters associated with malaria transmission in Korhogo, a city in northern Côte d’Ivoire. All data were collected over a 10-year period (2004–2013). Rainfall, temperature and Normalized Difference Vegetation Index (NDVI) were the climate and environmental variables considered. Association between these variables and clinical malaria data was determined, using negative binomial regression models. From 2004 to 2013, there was an increase in the annual average precipitation (1100.3–1376.5 mm) and the average temperature (27.2°C—27.5°C). The NDVI decreased from 0.42 to 0.40. We observed a strong seasonality in these climatic variables, which resembled the seasonality in clinical malaria. An incremental increase of 10 mm of monthly precipitation was, on average, associated with a 1% (95% Confidence interval (CI): 0.7 to 1.2%) and a 1.2% (95% CI: 0.9 to 1.5%) increase in the number of clinical malaria episodes one and two months later respectively. A 1°C increase in average monthly temperature was, on average, associated with a decline of a 3.5% (95% CI: 0.1 to 6.7%) in clinical malaria episodes. A 0.1 unit increase in monthly NDVI was associated with a 7.3% (95% CI: 0.8 to 14.1%) increase in the monthly malaria count. There was a similar increase for the preceding-month lag (6.7% (95% CI: 2.3% to 11.2%)). The study results can be used to establish a malaria early warning system in Korhogo to prepare for outbreaks of malaria, which would increase community resilience no matter the magnitude and pattern of climate change.

## Introduction

Malaria is a mosquito-borne infectious disease caused by parasitic protozoa of the genus *Plasmodium* that is transmitted by female *Anopheles* mosquitoes. Malaria transmission is strongly influenced by climatic and anthropogenic factors [1].

The efforts of the international community over the past 15 years have reduced malaria risk levels by 40% from 2000 to 2015, and large regions of Africa are now in a position to consider elimination strategies [2]. Despite this progress, about 3.2 billion people remain at risk of malaria in 2015 [3]. In 2015 alone, there were an estimated 214 million new cases of malaria and 438 000 deaths [3]. Approximately 90% of all malaria deaths occur in sub-Saharan Africa and most of them are in children under 5 years old [3].This is because the most effective malaria vector (i.e *Anopheles gambiae)* is the most widespread in Africa and the most difficult to control [4]. Malaria’s geographic range, seasonal pattern, and/or intensity of transmission are influenced by social and ecological systems and any change is likely to affect the epidemiology of malaria [1, 5–7]. Because of the association between weather and climate patterns, particularly temperature, precipitation, and extreme events, and malaria, climate change is expected to affect the distribution and seasonal activity of *Anopheles* mosquitoes, assuming no change in malaria control programs [8, 9].

From 1970 to 2000, West Africa experienced an increase in temperature, a decrease in precipitation, narrowing and shifting of the rainy season and extreme events such as floods [10–12]. Since 2000, a slight increase in precipitation was observed [13]. These changes affect ecosystem functioning and may contribute to the emergence or re-emergence of diseases, including vector-borne diseases such as malaria.

Rainfall and temperature affect the development of larvae in the aquatic environment and the survival of adult mosquitoes. An increase in rainfall increases the availability, persistence and dimensions of *Anopheles* larval habitats, although this depends on parameters such as local evaporation rates, soil percolation and slope of the terrain [5, 14]. Survival of vectors and parasites is temperature dependent, with too cold and too warm temperatures generally having a negative impact [15]. Some studies suggest that probabilistic seasonal climate forecasts can be used to predict malaria incidence in epidemic-prone areas [16–19]. But the influence of climatic parameters is complex and varies according to region and the ecology of the vectors [1, 20].

Malaria is endemic in Côte d’Ivoire and the estimated incidence of malaria in 2015 in the country was 330 cases per 1000 population [3]. In Côte d’Ivoire, there is considerable climatic variation from north to south, with three (3) different ecological zones [21]. Korhogo is the largest city of the northern part, where the climate regime is the Sudanian type, or semi-arid [21, 22]. This is also the driest region of the country. The average annual temperature is 27.0°C and the average annual precipitation is between 1,000 mm and 1,200 mm [23]. The vegetation has degraded over time and currently consists of dry forest and savanna [24, 25]. Korhogo experienced a severe drought in 2004 and 2005 that dried up several sources of drinking water, affecting nearly 200,000 people. The region also experienced heavy rains and floods in 2006 and 2007 [26]. This provides an interesting opportunity for studying effects of climatic variables (temperature, rainfall and vegetation cover) on malaria incidence.

Determining the spatial and temporal distributions of malaria are important planning interventions to target populations living in high-risk areas. Projections of possible future burdens with climate change can be used to further improve malaria control programs as the climate continues to change. Because this information is not available in northern Côte d’Ivoire, the purpose of this study was to analyze environmental parameters in relation to malaria transmission in Korhogo, based on statistical analyses of environmental parameters and clinical data, and remote sensing analysis of geospatial data over the period 2004–2013. In addition to investigating the role of meteorological parameters, we also analyzed associations between the Normalized Difference Vegetation Index (NDVI) and malaria to understand the potential impact of changing vegetation on the transmission of malaria. These analyzes can inform current and future malaria control measures in this region.

## Materials and methods

### Ethics statement

This research was carried out in the frame of a project entitled **“Vulnerability and resilience to malaria and schistosomiasis in the northern and southern fringes of the Sahelian belt in the context of climate change”** funded by TDR/WHO and the International Development Research Centre of Canada (IDRC) and implemented from 2013 to 2017 in Côte d’Ivoire and Mauritania. In Côte d’Ivoire, the National Ethics Committee cleared the research protocol under the reference N° 10056/MSHP/CNER-dkn, dated 29 May 2013. In addition, a letter of support and agreement was obtained from the Director of the health district of Korhogo. At the beginning of the project, a workshop was organized with local decision makers, health professional and communities to discuss and agree on the objectives of and methods used in the project.

### Study area

Korhogo is located in the north of Côte d'Ivoire (Longitude 05° 38'19'' west and Latitude 09°27'41''North). In 2014, the population was estimated at 286,071 inhabitants [27]. It is the capital of the Poro region, which covers an area of approximately 12,500 km^2^. This area is located in the south part of the Sahelian band. Korhogo is some 600 km north of Abidjan, the economical capital. The seasons are controlled by the movement of the Intertropical Convergence Zone (ITCZ) [28].

### Clinical data

Clinical malaria cases were collected from health facilities using the patient consultation register. The registers were searched by trained health workers (nurses) recruited locally. We were looking for the longest continuous series in malaria counts. Finally, among the 15 health centres of the city of Korhogo and surroundings, four had complete data during the decade 2004–2013. Three centres were exclusively urban (1, 3, and 4) and one (2) peri-urban. Malaria cases were anyone with fever or fever with headaches, back pain, chills, sweats, myalgia, nausea or vomiting, who were clinically diagnosed as having malaria or whose disease was confirmed by laboratory diagnosis.

### Climate data

The meteorological parameters considered were precipitation and temperature. These data were collected from the Korhogo airport station of SODEXAM (Société d'Exploitation et de Développement Aéroportuaire Aéronautique et Météorologique) that manages all the weather network stations of Côte d'Ivoire. In Côte d’Ivoire, there is one weather station per secondary city. Thus, the climatic data are from the station of Korhogo. Data are collected, treated and saved by SODEXAM from which we obtained the data in Excel format. Temperature was recorded in degree Celsius (°C) and precipitation in millimeter (mm). Data were recorded monthly over the period 2004–2013.

### Remote sensing data

A time series of the NDVI covering the years 2004–2013 was used. NDVI is a measure of the status of the photosynthetic green vegetation which can protect mosquito breeding sites. NDVI version 5 data were obtained in 1 km resolution from the Moderate Resolution Imaging Spectroradiometer (MODIS) satellite images. Data were downloaded from the NASA Land Processes Distribution Active Archive Center (2011) using the data library of International Research Institute for Climate and Society (IRI) of Columbia University in New York, USA [29]. Data were averaged across all grid cells covering the area of Korhogo.

### Statistical analysis

Time series as well as associations between monthly malaria counts and meteorological parameters or NDVI were studied for the period 2004–2013.

Statistical analyses were conducted using STATA version 14 (Stata Corporation; College Station, USA). Scatter plots smoothed using the algorithm LOWESS (locally weighted scatter plot smoothing), which is an outlier resistant method based on local polynomial fits, were used to explore the patterns of malaria cases [30]. The distributions of yearly and monthly means of temperature, rainfall, NDVI and malaria cases (seasonality) were described using boxplots [31], histograms and line graphs.

The associations between environmental parameters (precipitation, temperature and NDVI) and malaria cases were studied using negative binomial regression models taking into account the over-dispersion present in the residuals [32]. We modeled the expected value E(Y) of monthly malaria case counts Y by health center as an exponential function of a linear predictor defined by the scalar product of a column vector X of independent variables (including environmental parameters) and a row vector β of associated regression coefficients, i.e.,

E(Y)=exp⁡(βX)

where exp() is the natural exponential function.

The variables precipitation, temperature and NDVI in the month of the malaria case and in the three preceding months were included as explanatory variables in the regression models. This was done by putting a linear constraint on the coefficients of the different lags (i,e, the coefficients b_0_, b_1_, b_2_ and b_3_ of each of these variables at lags 0, 1, 2 and 3, respectively, were supposed to be of the form b_i_ = c_0_+c_1_·i+c_2_·i^2^) [33].

Because there were different time trends of malaria cases between urban and rural centers and to a lesser degree also across the urban centers we also included center-specific non-linear time trends along with fixed center intercepts into the model. These were defined as cubic polynomials of the month of observation (which ranged from 1 to 120).

To remove some remaining positive autocorrelation, we also included the Pearson residual of the previous month into the model. As meteorological variables are correlated, we also subjected them to a principal component analysis and used the first principal component and its values in the three preceding months as predictor variables of monthly malaria incidence. The results are presented in Tables A, B and C of S1 File.

The geographical distribution of malaria was studied by mapping the studied health centres. Maps were produced using ArcView version 10 (Environmental Systems Research Institute Inc; Redlands, USA).

## Results

### Distribution of malaria cases

**Fig 1:**
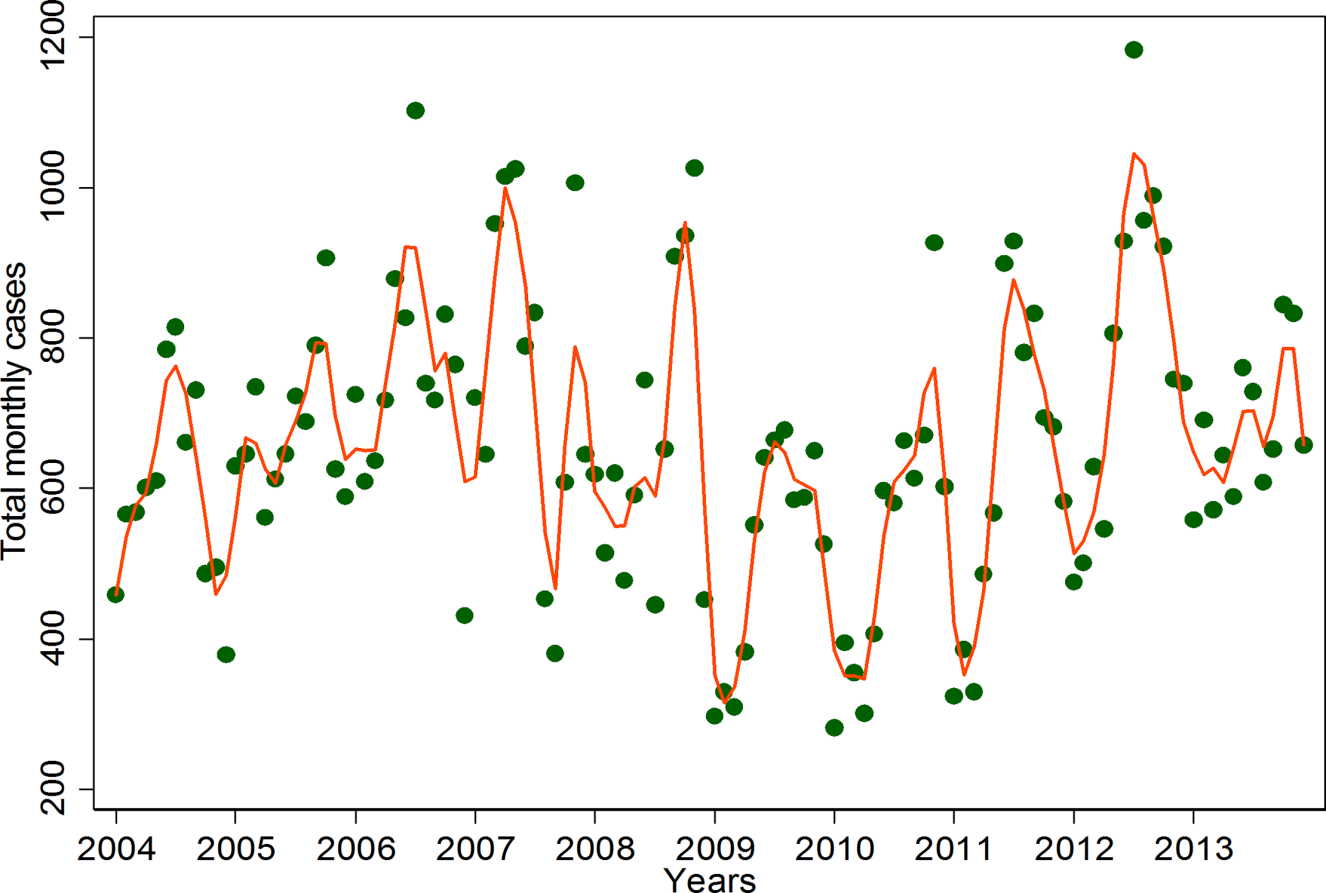
Time series of monthly reported malaria cases from 2004 to 2013. The dots represent monthly counts of malaria cases attending the four facilities while the line has been obtained using a lowess smoother with a bandwith of 0.05.

Fig 1 shows a strong variation in clinical malaria cases over years. The highest numbers of cases were observed in 2007 and 2012. The lowest numbers of cases were in 2005, 2009 and 2010.

**Fig 2:**
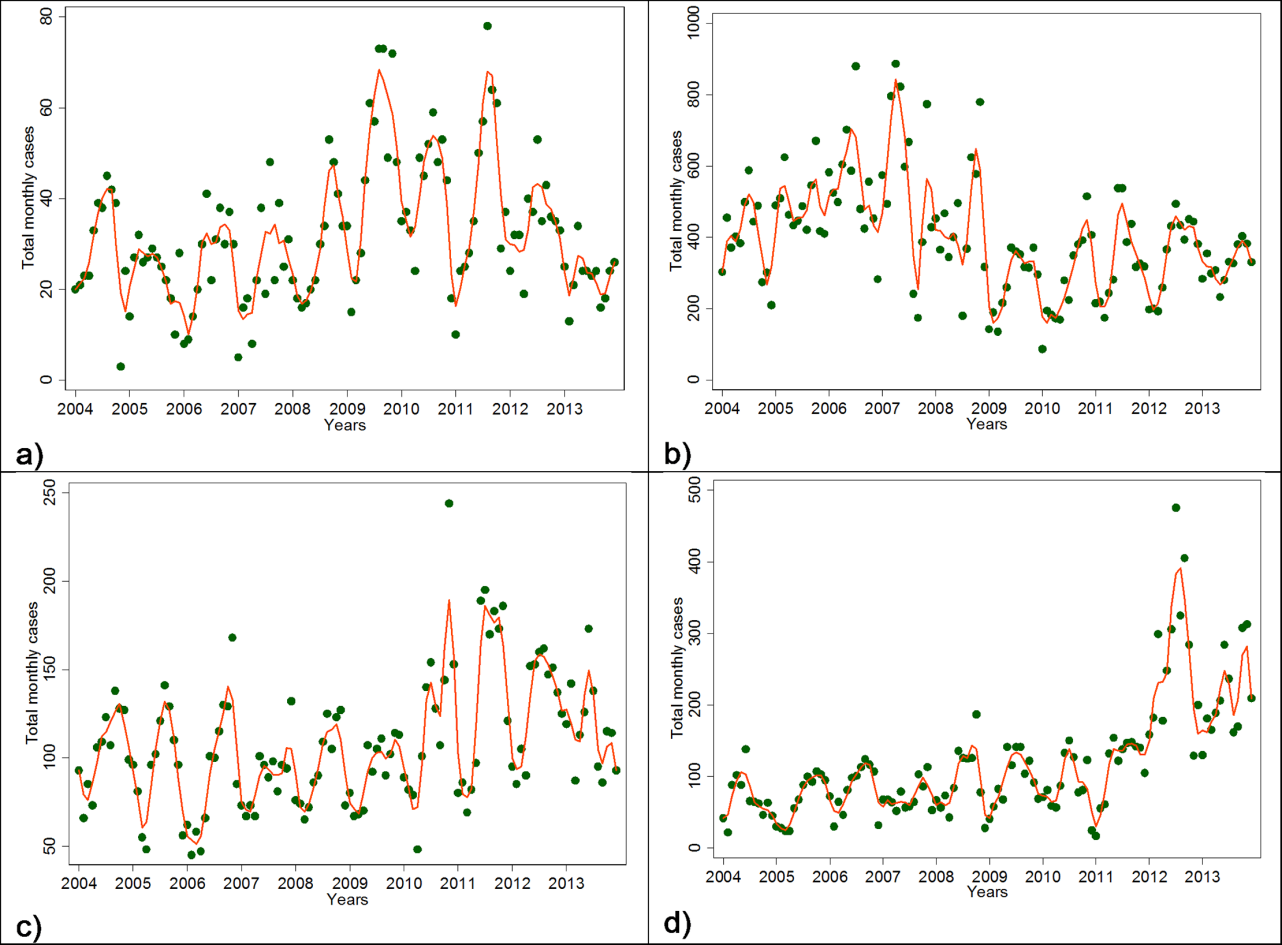
Time series of monthly reported malaria cases per health centre. a) centre 1 = CSI AN NOUR, b) centre 2 = HB TORGO, c) centre 3 = IP SOBA, d) centre 4 = IP TENEMANGA. The dots represent monthly counts of malaria cases attending the respective facility while the lines were obtained using a lowess smoother with a bandwith of 0.05.

Fig 2 shows that the time series of monthly malaria incidence varied significantly from year-to-year. In health centres 1, 3 and 4, cases increased over the time series, but declined in centre 2. Our models of malaria incidence therefore included center-specific time trend functions.

**Fig 3:**
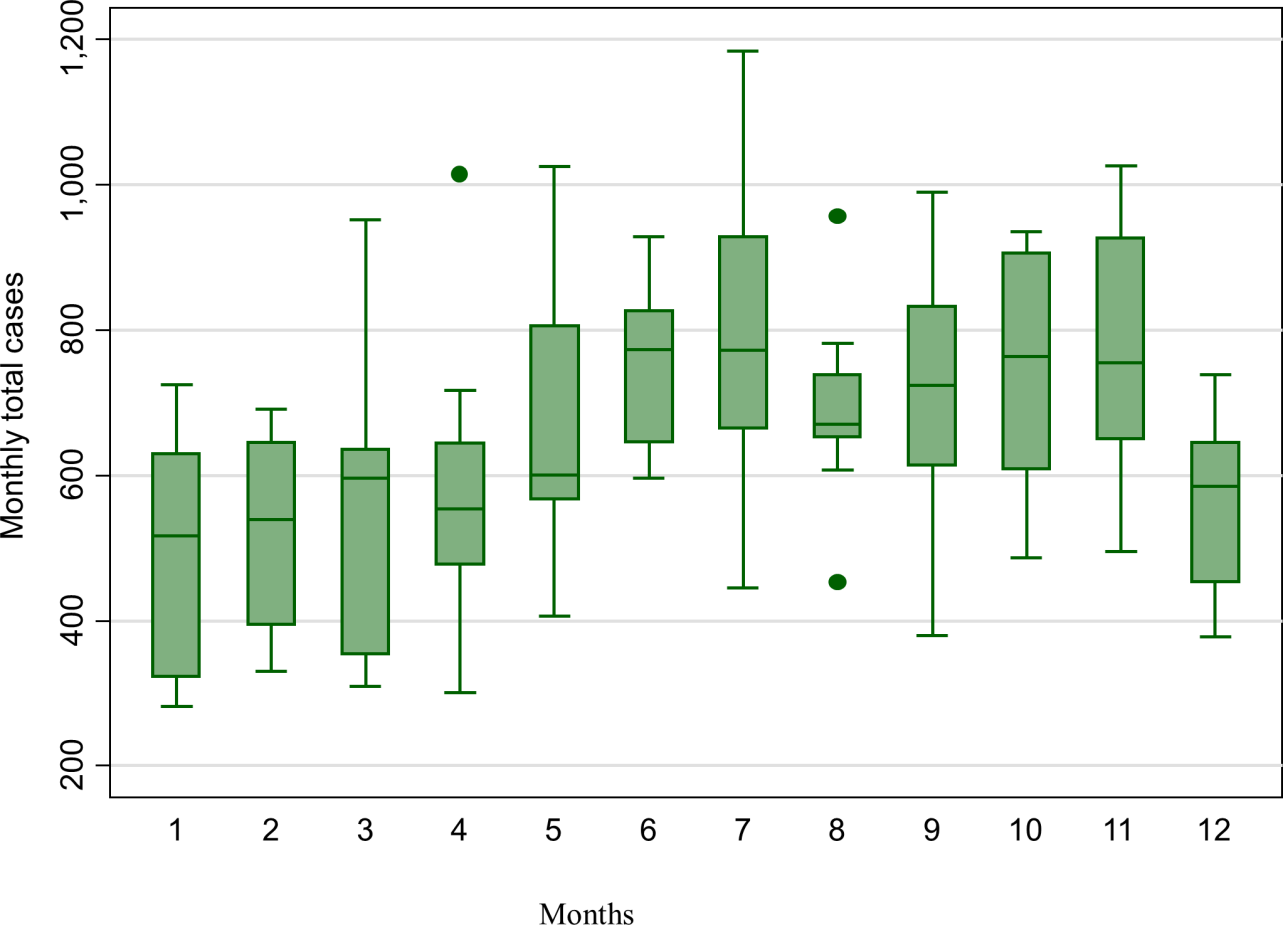
Distribution of monthly malaria counts across the years 2004–2013 in Korhogo.

Fig 3 shows the distribution of monthly mean malaria counts over the years 2004 to 2013. The highest numbers of cases occurred starting from June to November. The lowest malaria counts appeared from December to April.

### Climate and environmental parameters and malaria incidence

#### Temporal distribution of rainfall and malaria cases in Korhogo

**Fig 4:**
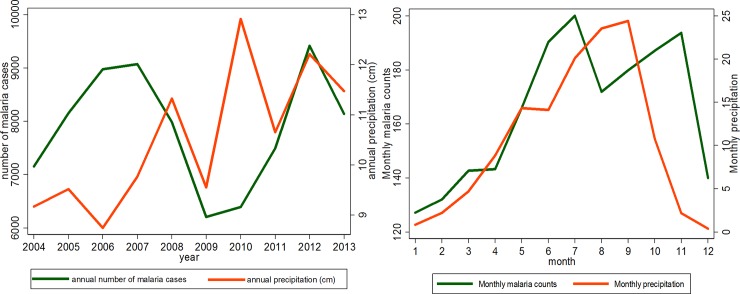
Time series of annual (Fig 4A) and average monthly (Fig 4B) rainfall and malaria cases over the period 2004–2013.

Fig 4 depicts the time series of annual (Fig 4A) and average monthly (Fig 4B) rainfall as well as malaria count for 10 years (2004–2013) in Korhogo. Precipitation varies across the years, with peaks in 2008, 2010 and 2012. The first peak of numbers of malaria cases was observed in 2007, following by a decrease until 2009 but remain high in 2008 where there was a first peaks in rainfall. The numbers of cases increase from 2010 up to second high values in 2012. Then, in general, the highest numbers of malaria cases were observed when precipitation increased.

Average monthly rainfall showed a strong seasonal cycle. The highest rainfall values were observed from May to September when average rainfall was more than 150mm/month and the lowest values were from October to April with average rainfall below 150 mm. This results show that the region of Korhogo is characterized by two main seasons, a dry season from October to April and a rainy season from May to September. We noted that the trend of monthly malaria distribution overlap the seasonal cycle of rainfall.

#### Temporal distribution of temperature and malaria cases in Korhogo

**Fig 5:**
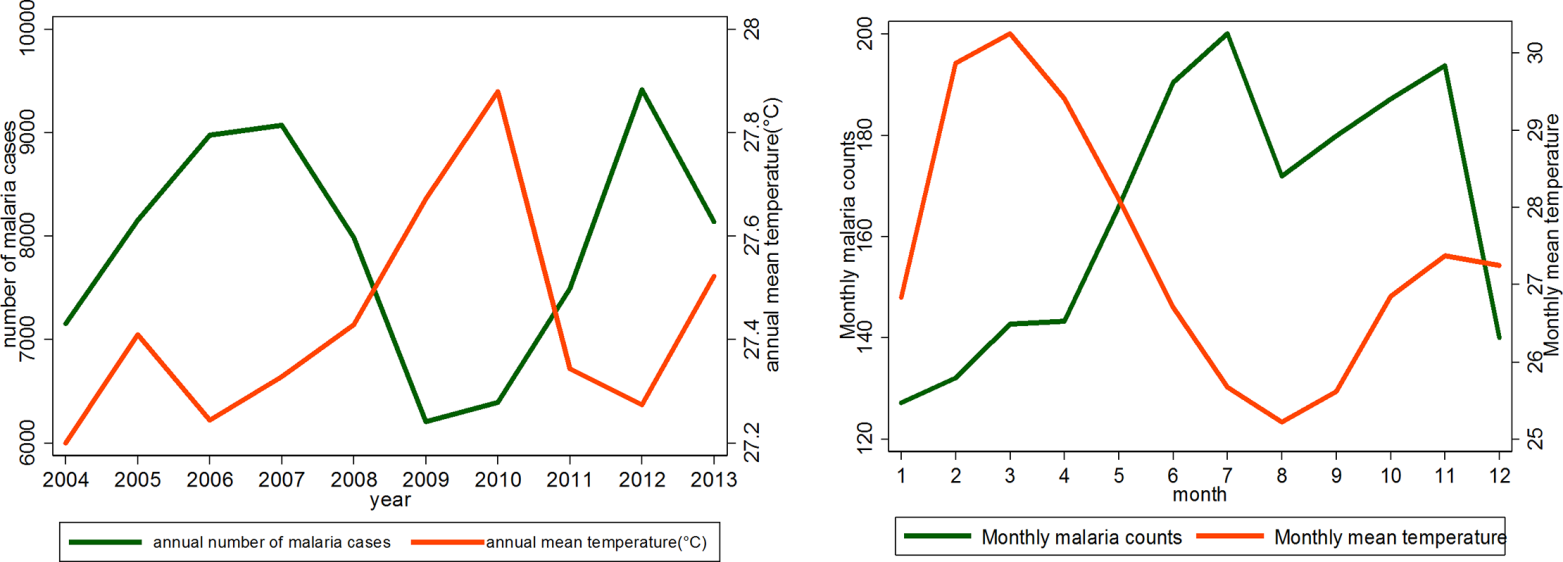
Time series of average annual (Fig 5A) and monthly (Fig 5B) temperature and malaria cases over the period 2004–2013.

Fig 5 shows the time series of average annual (**Fig 5A**) and monthly (**Fig 5B**) temperature and malaria over the 10 years (2004–2013). It indicates an increase in annual average temperature from 27.2°C to 27.5°C. The highest annual mean temperature, 27.9°C, was in 2010 (corresponding to a lowest value in malaria count), followed by a drop until 27.3°C in 2012. This drop in temperature was opposed to an increase in malaria incidence that reached its peak in 2012. The monthly mean temperatures show a clear seasonal cycle. The period November to May is characterized by high temperatures, with monthly means above 27.0°C. The period from June to October experiences mean temperatures below 27.0°C. The detailed temperature time series from year to year show that the period from November to April experienced average temperatures between 25.0°C and 30.0°C. The remaining months experienced more moderate temperatures and lowest temperatures in August and September. This seasonality of the temperature is contrasting the seasonality of malaria incidence.

#### Temporal distribution of NDVI and malaria cases

**Fig 6:**
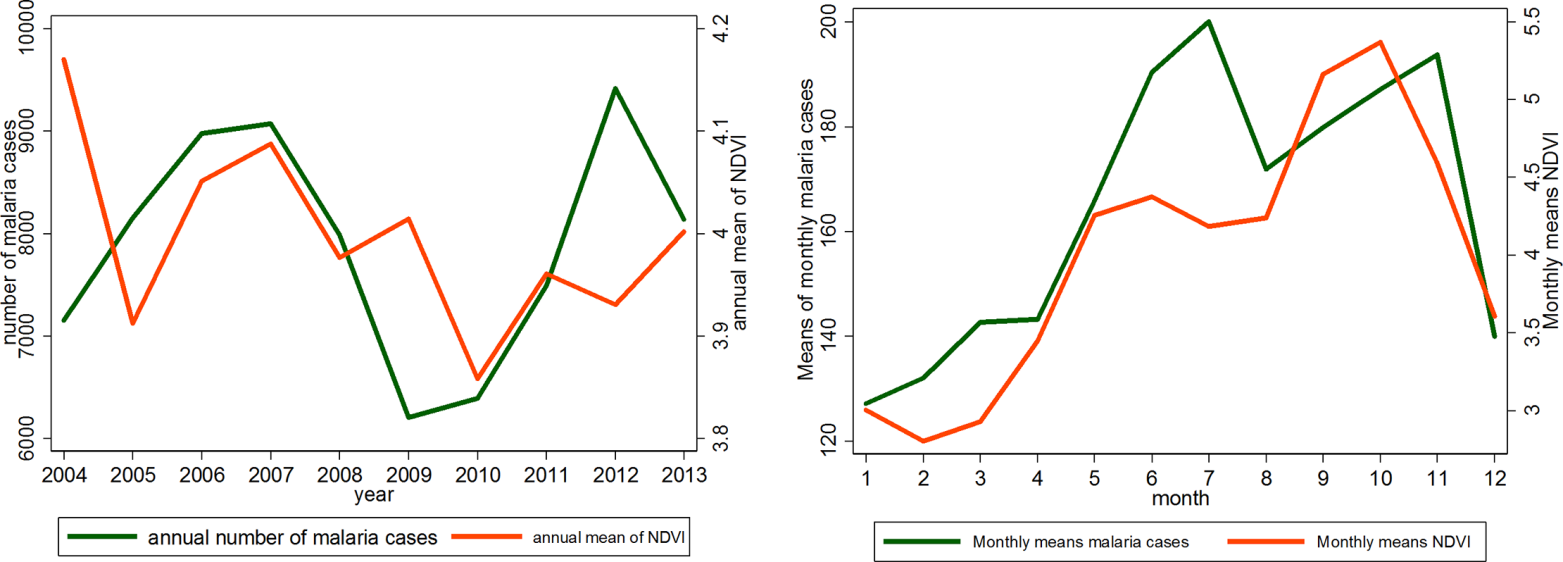
Annual means of NDVI and malaria cases over 2004–2013.

Fig 6 shows that the annual average NDVI decreased over the period 2004 to 2013 and the distribution parallels that of malaria incidence. Moreover, the seasonality of NDVI overlap that of malaria incidence. Both, monthly mean NDVI and monthly malaria cases increase during the rainy season from May to November.

### Association between climate, environmental parameters and malaria distribution

Table 1 shows that each 1 cm increase in rainfall during the previous and the prior two months were on average associated with a 1% (95% CI: 0.7 to 1.2%) and an 1.2% (95% CI: 0.9 to 1.5%) increase in monthly malaria incidence respectively. Moreover, each 1°C increase in monthly temperature was on average associated with a 3.5% (95% CI: 0.1 to 6.7%) decrease in malaria incidence. In addition, each 1°C increase in temperature during the previous and the prior two months were on average associated with a 4.2% (95% CI: 2,1 to6.2%) and a 2.1% (95% CI: 0.3 to 3.9%) decrease in malaria incidence respectively. A 0.1 unit increase in monthly NDVI was associated with an 7.3% (95% CI: 0.8 to 14.1%) increase in the monthly malaria count. There was a similar increase for the preceding-month lag (6.7% (95% CI: 2.3% to 11.2%)).

**Table 1:** Estimated effects of climatic variables across different months on the monthly malaria count.

TOTAL_Malaria	Incidence Rate Ratio	p-value	[95% Conf.Interval]	
Mean T_of the month	0.965	0.04	0.933	0.999
Mean T_ previous month	0.958	<0.0001	0.938	0.979
Mean T_two months before	0.979	0.02	0.961	0.997
P_ of the month	0.997	0.15	0.992	1.001
P_ previous month	1.01	<0.0001	1.007	1.012
P_ two months before	1.012	<0.001	1.009	1.015
NDVI_of the month	1.073	0.03	1.008	1.141
NDVI_ previous month	1.067	0.002	1.023	1.112
NDVI_ two months before	0.999	0.97	0.957	1.043

Models included an indicator variable for center, a linear term of year and the respective mean value (i.e., of temperature, quantity of rain fall or NDVI) at the same month, the month before and two months before.

Additional related results are given in Tables B and C of S1 File and health center-specific scatter plots of observed and fitted monthly malaria counts according to the model underlying Table 1 are combined in S4 Fig.

## Discussion

This study analyzed the change in temperature, precipitation and NDVI parameters in Korhogo and the influence of these changes on the transmission of malaria from 2004 to 2013. West Africa is a region where climate variability is most pronounced [13]. We found (Figs 4–6) large inter annual variation in rainfall, temperature and NDVI over the period 2004–2013. Our study documents seasonal cycles in the distribution of monthly rainfall, temperature and vegetation cover defining two climatic seasons, a rainy (May to September) and a dry season (October to April), in Korhogo. These results are consistent with the studies of Goula *et al* (2007) [22] and Kouakou *et al* (2014) [23] in the region. Rainfall patterns in West Africa are linked to the seasonal movement of the inter-tropical convergence zone. These seasonal cycles influence the spatial distribution of malaria [34].

In this study, the seasonal variation in the number of malaria cases parallels the seasonality of rainfall and NDVI and is opposite to the seasonality of temperature. These variations were expected based on understanding of the roles of rainfall and temperature in the life-cycle of *Anopheles* and the transmission cycle of the malaria parasite [35, 36]. Studies have already shown that high or low temperatures limit the activities of mosquitoes and the development of Anopheles larvae [1, 36, 37]. The department of Korhogo represents the most arid zone of Côte d’Ivoire with a long and warm dry season. During this season, the number of breeding sites is reduced and air and water temperatures are very high. We can therefore understand that rises in air temperature can reduce the activity of anopheles larvae and thus the transmission of malaria in the following months. The transmission rate mostly depends on rainfall variation and the distribution of more specific environmental factors, potential breeding sites, such as potholes, small pits, cans or bins. Another explanatory factor might be the extension of the city into new neighborhoods and the increase in urban agriculture. That may contribute to an increase the number of breeding sites and thus, malaria transmission. In fact, the population of the city increased from 170,730 inhabitants in 2004 to 272,118 inhabitants in 2013 [27]. The role of those environmental factors in disease transmission may explain the difference shown by Fig 2 of the time series of monthly malaria incidence between exclusive urban health centers (centers 1, 3 and 4) visited mostly by urban dwellers living in the surrounding areas and the peri-urban center of Torgo (Center 2), visited by urban and peri-urban dwellers. The duration of the rainfall season is also important. In regions where temperature is high but rainfall is limiting, such as the fringes of the North African deserts, mosquito populations increase rapidly at the onset of rain because of short developmental cycles. Consequently, three months of rain would be sufficient to constitute one transmission season [38]. However, where temperature is limiting during the colder season, mosquito populations increase slowly at the onset of rain, with gradually rising temperatures, owing to long developmental cycles. Our results show that monthly malaria incidence was positively associated with the quantity of rain one and two months before and the vegetation cover of the same and the previous month (Table 1). If vegetation cover was not included in the model, quantity of rainfall in the preceding months was positively associated with malaria incidence. This association changed after inclusion of vegetation cover. This finding might reflect the fact that NDVI is positively correlated with preceding rainfall [24, 39, 40]. This finding is also consistent with other publications where NDVI was strongly related to the incidence of malaria particularly in Sahelian or Sudanese savannah environments [41–44]. This suggests an early warning system can be developed to forecast the start of the malaria season using temperature and precipitation forecasts. Coupled with public health interventions to prepare for increased malaria transmission, including vector control programs, such an early warning system can reduce the burden of malaria. Further research is needed using, for example satellite images, to better characterize land cover, grass species and their correlation with breeding site spatial distribution [45, 46]. Developing an early warning system could reduce the burden of malaria in Korhogo.

## Limitations

The biggest limitation was availability of clinical data. We expected to have a long series of clinical malaria cases but we encountered two types of weakness in the health facilities.

-There were no registers for certain periods;
-Some registers had lot of missing information.

The military and political crisis experienced by Côte d'Ivoire from 2002 to 2011 caused the destruction of health records. The missing data were also due to some health personnel not collecting all patients’ data during consultations. Fortunately, 10 years (2004–2013) of continuous clinical malaria data in four health facilities were available for study. As there was no laboratory confirmation of malaria, the data must have been contaminated with a certain proportion of cases of fever unrelated to malaria. If this proportion varied over the year it may have led to some bias in our effect estimates for the climatic variables. Another limitation might be that we could not take into account fluctuations in the population due to migration or the introduction of preventive measures, although these effects may have been partly captured by the use of center-specific non-linear time trends.

## Conclusion

Korhogo experiences a strong seasonality in the distribution of monthly rainfall, temperature and vegetation cover that define its two climatic seasons. The seasonal distribution of malaria cases over the decade 2004–2013 is coherent with this seasonality. Malaria incidence showed a negative association with the temperature of the same month and a positive one with the quantity of rainfall two months before. These results can be used to develop an early warning system to forecast periods of high infection risk.

Knowing how climate and environmental factors affect malaria transmission dynamics is an important first step in developing policies and programs to prepare for changing weather patterns associated with climate change; further increases in temperature and precipitation would be expected to increase the burden of malaria without additional interventions. Moreover, there is a need to increase the number of well-trained health professionals being sensitized to the importance of health records for health planning and research. Ongoing capacity building for actual health professionals is also needed.

## Supporting information

S1 Fig Time series of average annual (a) and monthly (b) temperature over the period 2004–2013.
(TIF)

S2 Fig Rainfall anomalies over 2004–2013 in Korhogo. (TIF)

S3 Fig Monthly mean NDVI in Korhogo over 2004–2013 (USGS LandDAAC MODIS version_005 WAF). (TIF)

S4 Fig Plot of the empirical monthly malaria counts against counts predicted, by centre. a) centre 1 = CSI AN NOUR, b) centre 2 = HB TORGO, c) centre 3 = IP SOBA, d) centre 4 = IP TENEMANGA.
(TIF)

S1 File Supplementary material_M’Bra et al. (docx-file). **—Table A.** Rank correlations of the three climatic variables considered; **Table B.** Estimated associations of the monthly malaria count with the climate scores of the same and the two preceding months.; **Table C.** Estimated associations of the monthly malaria count with concurrent and preceding monthly levels of each climatic variable considered in a separate model.
(DOCX)

S2 File Climate data M’Bra et al. (xlsx-file). Contains variables year, month, temp (= mean monthly temperature in°C), rainfall (= monthly quantity of rain fall in mm), ndvi (= monthly value of normalized difference vegetation index).
(XLSX)

S3 File Clinical data M’Bra et al. (xlsx-file). Contains variables health_centre, year, month, malaria_under5 (= monthly count of malaria cases among children under 5 years), malaria_over5 (= monthly count of malaria cases among persons aged 5 years or more), malaria_total (= monthly count of malaria cases).
(XLSX)
