# Supplementary figures and images for: Impact of climate variability on the transmission risk of malaria in northern Côte d'Ivoire
Source: PLoS One. 2018 Jun 13;13(6):e0182304. doi: 10.1371/journal.pone.0182304 (PMC5999085; doi:10.1371/journal.pone.0182304)

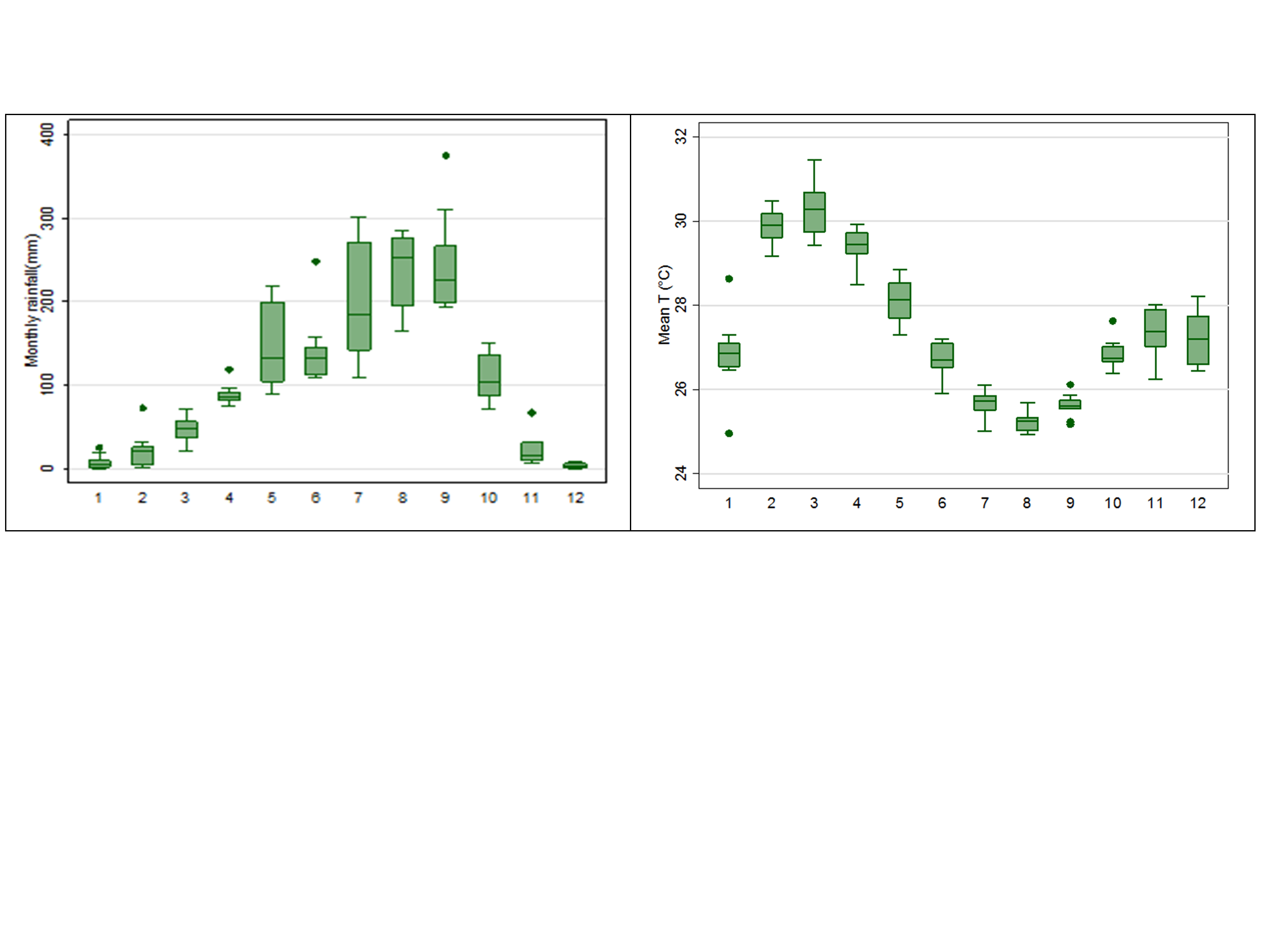

Supplement: S1 Fig — Time series of average annual (a) and monthly (b) temperature over the period 2004–2013. (TIF) [file pone.0182304.s001.tif]

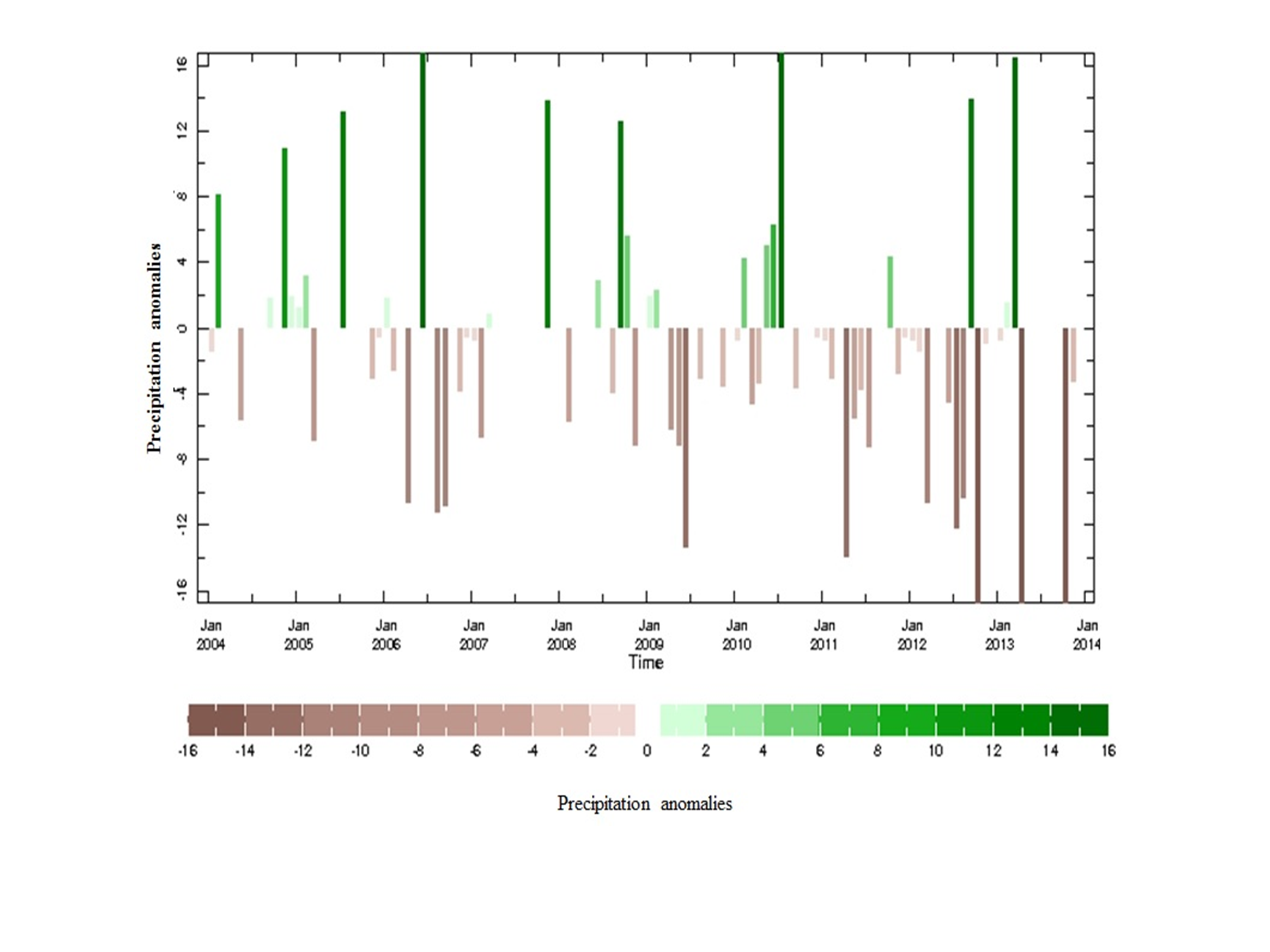

Supplement: S2 Fig — (TIF) [file pone.0182304.s002.tif]

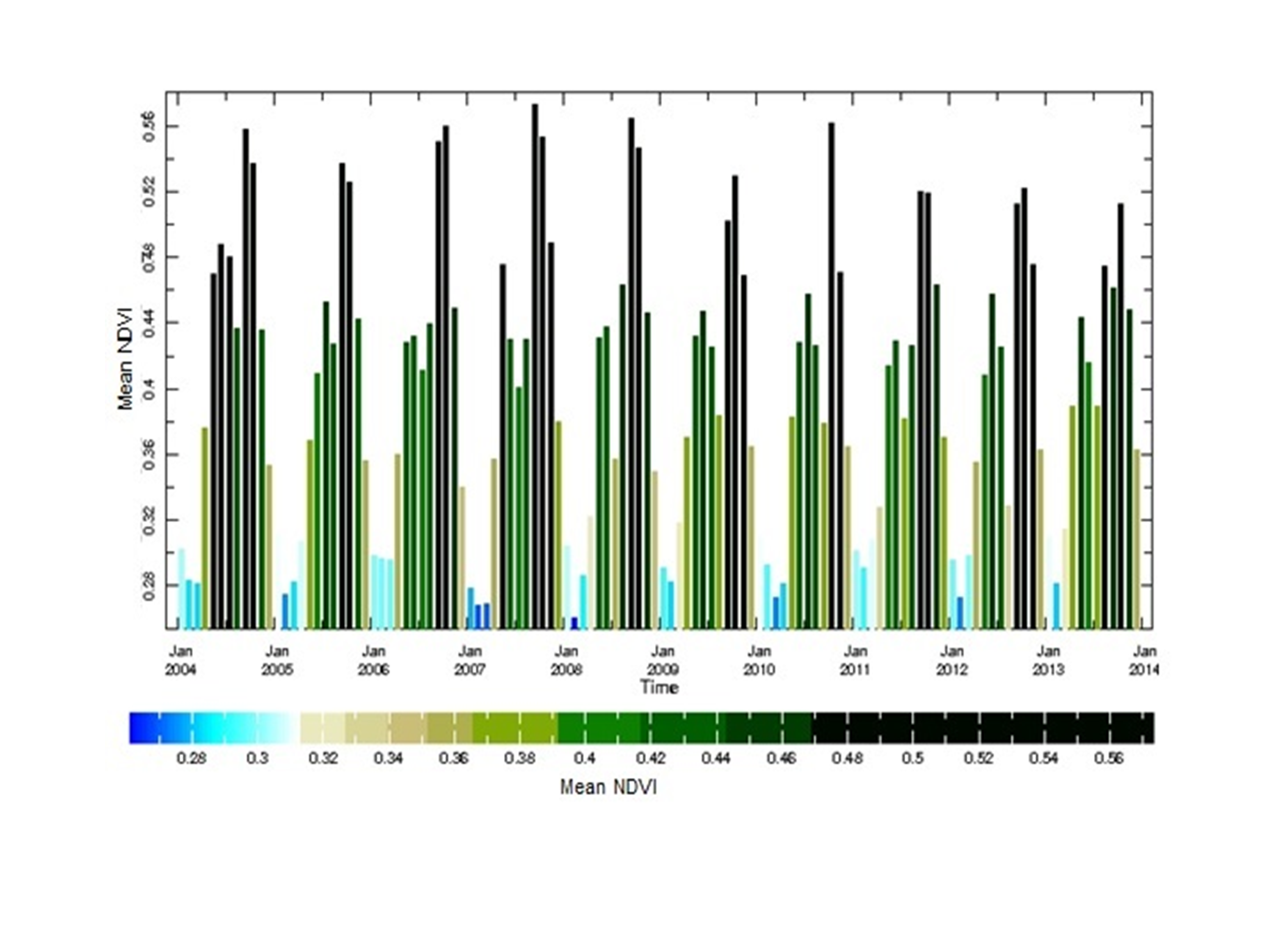

Supplement: S3 Fig — (TIF) [file pone.0182304.s003.tif]

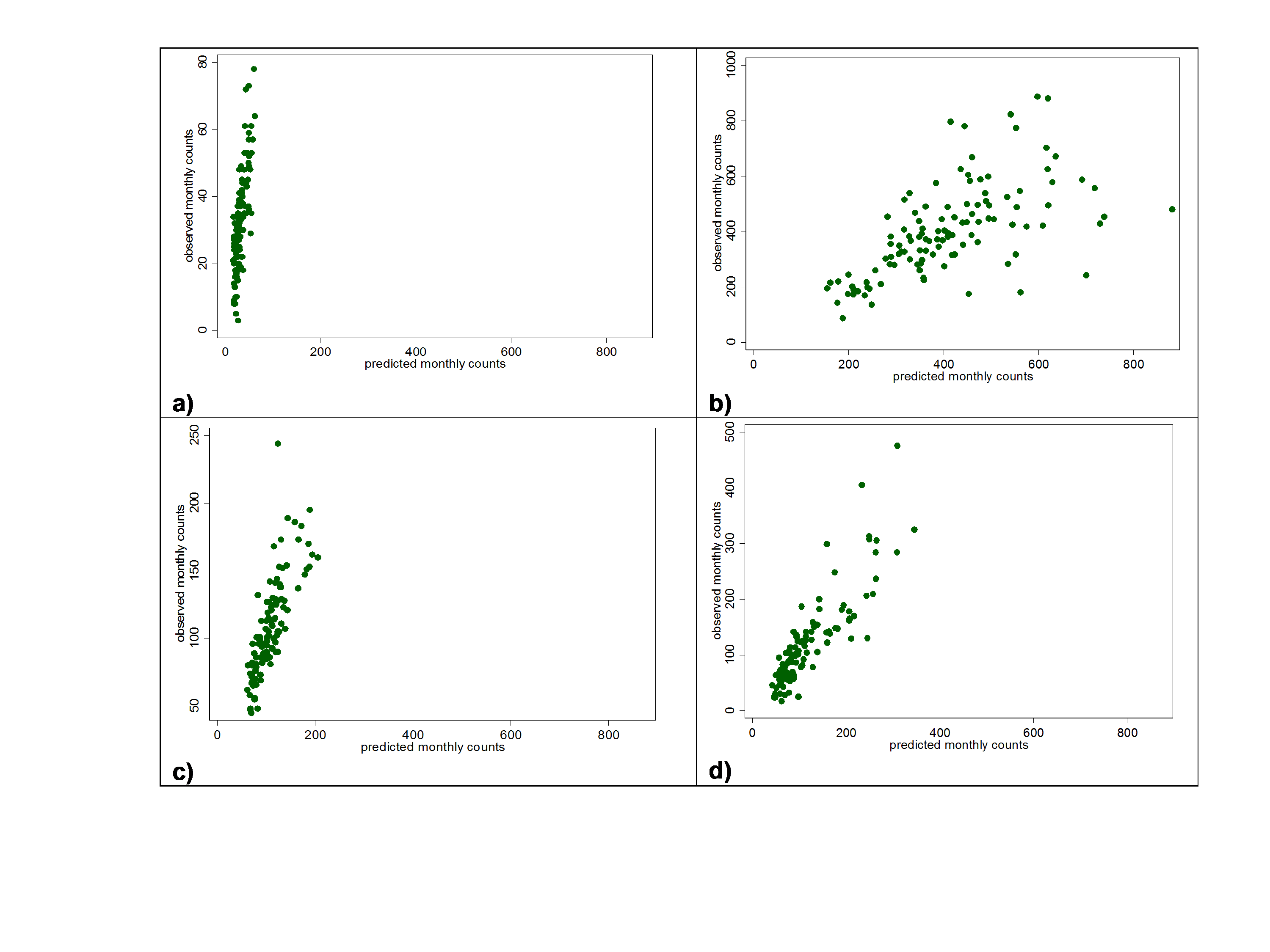

Supplement: S4 Fig — a) centre 1 = CSI AN NOUR, b) centre 2 = HB TORGO, c) centre 3 = IP SOBA, d) centre 4 = IP TENEMANGA. (TIF) [file pone.0182304.s004.tif]
