# Supplementary material for: Impact of climate variability on the transmission risk of malaria in northern Côte d'Ivoire
Source: PLoS One. 2018 Jun 13;13(6):e0182304. doi: 10.1371/journal.pone.0182304 (PMC5999085; doi:10.1371/journal.pone.0182304)
Supplement: S1 File — —Table A. Rank correlations of the three climatic variables considered; Table B. Estimated associations of the monthly malaria count with the climate scores of the same and the two preceding months.; Table C. Estimated associations of the monthly malaria count with concurrent and preceding monthly levels of each climatic variable considered in a separate model. (DOCX) [file pone.0182304.s005.docx]

**Impact of climate variability on the transmission risk of malaria in northern Côte d’Ivoire**

**Supplementary material**

**1. Study area**

S1 Fig shows the geographical distribution of the health facilities in which data were collected. Data were collected in three urban (in green on the map) and one peri-urban centres (in green below the map).

**S1 Fig: Study area and distribution of the health facilities in Korhogo**

**2. Seasonality of meteorological parameters**

S2 Fig presents the distribution of monthly rainfall (a) and monthly mean temperature (b). These graphs show a clear seasonal cycle of meteorological parameters.

**S2 Fig: Time series of average annual (a) and monthly (b) temperature over the period 2004–2013**

S3 Fig presents rainfall anomalies over the period 2004-2013 calculated using the formula:

${\boldsymbol{r}\boldsymbol{'}}_{\boldsymbol{ij}}\boldsymbol{=}\boldsymbol{r}_{\boldsymbol{ij}}\boldsymbol{-}\frac{\boldsymbol{1}}{\boldsymbol{N}_{\boldsymbol{i}}}\sum_{\boldsymbol{j}\boldsymbol{=}\boldsymbol{1}}^{\boldsymbol{N}} \boldsymbol{r}_{\boldsymbol{ij}}$ (1)

where:

***r’_ij_*** **=** monthly anomaly of rainfall, ***r_ij_* =** observed monthly quantity of rainfall, (**i =** month, **j =** year), and the remainder of the equation is the mean quantity of rainfall in month i over the period 2004-2013. The monthly anomaly thus represents the deviation of the quantity of rainfall in the respective month and year from the corresponding average monthly level over the period 2004-2013.

For most years of the 10 years’ series, the quantity of rainfall between June and November exceeded the average rainfall of the year (rainfall anomalies between 4 and 16 mm/month); with the exception of 2009 and 2013. Moreover, in 2006 and 2012, a high quantity of rainfall (rainfall anomalies = 16 mm/month) was observed only in June and August, respectively. The remaining periods remainders of the years were dry with rainfall anomalies less than

- 1mm/month.

**S3 Fig: Rainfall anomalies over 2004–2013 in Korhogo**

As shown on S4 Fig, there was a very low vegetation cover (NDVI <0.3) in January-February-March (blue) across all years. In April and December, the level of vegetation cover was low, with NDVI between 0.32 and 0.38 (yellow). The highest vegetation cover (NDVI > 0.4) was observed from May to November (green) with the peaks in September or October.

**S4 Fig: Monthly mean NDVI in Korhogo over 2004-2013 (USGS LandDAAC MODIS version_005 WAF).**

**3. Correlation between climatic variables and their impact on malaria transmission**

Correlation analysis showed that temperature was negatively correlated with rainfall (r= -0.57) and ndvi (r=-0.62). Moreover rainfall was positively correlated with ndvi (r=0.49). This indicates that rainfall led to an increase in vegetation cover.

**Table A:** Rank correlations of the three climatic variables considered

|  | Temperature | Precipitation | NDVI |
| --- | --- | --- | --- |
| Temperature | 1 |  |  |
| Precipitation | -0.566 | 1 |  |
| NDVI | -0.602 | 0.495 | 1 |

Subsequently, the three variables were subjected to a principal component analysis and the first principal component was used as a climate score variable. This score was positively correlated with rainfall (r = 0.67) and vegetation cover (r = 0.70) and negatively with temperature (r = -0.76). Its values are high in months with strong rainfall, high vegetation cover and low temperatures and low in hot and dry months with low vegetation cover. Increases in this score by one standard deviation one and two months before were associated with 15.9% (95% CI: 12.1 to 19.9%) and 9.7% (95% CI: 6.1 to 13.5%) increases in the monthly malaria count, respectively. In contrast, there was a negative association between monthly malaria incidence and the value of this score three months before.

**Table B:** Estimated associations of the monthly malaria count with the climate scores of the same and the two preceding months.

| TOTAL_Malaria | Incidence Rate Ratio | p-value | [95% Conf.Interval] | |
| --- | --- | --- | --- | --- |
| climate score of the month | 1.038 | 0.13 | 0.989 | 1.089 |
| climate score previous month | 1.159 | <0.0001 | 1.121 | 1.199 |
| climate score two months before | 1.097 | <0.001 | 1.061 | 1.135 |

- **Model with one climatic variable at a time**

We also ran analyses with one climatic variable at a time represented by the monthly means of the same and the two preceding months and compared the AIC’s of the three models. The lowest AIC was found for the model with NDVI, the second lowest for the model with rainfall and the highest for the model with temperature alone.

**Table C:** Estimated associations of the monthly malaria count with concurrent and preceding monthly levels of each climatic variable considered in a separate model

| TOTAL_Malaria | Incidence Rate Ratio | p-value | [95% Conf.Interval] | | AIC |
| --- | --- | --- | --- | --- | --- |
| Mean T_of the month | 0.963 | 0.002 | 0.940 | 0.986 | 4548 |
| Mean T_ previous month | 0.932 | <0.0001 | 0.932 | 0.963 |  |
| Mean T_two months before | 0.974 | 0.002 | 0.959 | 0.990 |  |
| P_ of the month | 1.001 | 0.52 | 0.997 | 1.005 | 4517 |
| P_ previous month | 1.012 | <0.0001 | 1.009 | 1.014 |  |
| P_ two months before | 1.011 | <0.0001 | 1.009 | 1.014 |  |
| NDVI_of the month | 1.136 | <0.0001 | 1.090 | 1.184 | 4498 |
| NDVI_ previous month | 1.115 | <0.0001 | 1.085 | 1.145 |  |
| NDVI_ two months before | 1.038 | 0.007 | 1.010 | 1.066 |  |

**4. Formulation of the model of monthly malaria counts and plot of the empirical monthly malaria counts against counts predicted by this model**.

The underlying negative binomial regression model included temperature, quantity of rain fall and NDVI of the same month and the three preceding months along with a separate cubic time trend function for each of the four health centers. Models also contained autoregressive terms to control residual serial.

The expected monthly count E(Y_i,t_) of malaria cases at facility i (i=1,2,3,4) in month t, conditional on temperature, rainfall and NDVI of the same and previous months and on past monthly counts of malaria cases at the same facility, was modeled as follows:

$$\ln\left( E\left( Y_{i,t} \right) \right)= \beta_{0}+ \sum_{i=1}^{4} \sum_{j=0}^{3} \beta_{1ij}C_{i}t^{j}+ \sum_{j=0}^{2} \beta_{2j}\sum_{l=0}^{3} l^{j}T_{t-l}+ \sum_{j=0}^{2} \beta_{3j}\sum_{l=0}^{3} l^{j}R_{t-l}+ \sum_{j=0}^{2} \beta_{4j}\sum_{l=0}^{3} l^{j}N_{t-l}+\beta_{5}r_{i,t-1}$$

where

Ci = indicator variable for health facility i (i = 1,2,3,4)

Yi,t = observed monthly count of malaria cases at facility i (i = 1,2,3,4) in month t

Tt = average temperature in month t

Rt = quantity of rainfall in month t

Nt = NDVI in month t

ri,t = Pearson residual (Yi,t – E(Yi,t)) / √E(Yi,t) at facility i in month t

A negative binomial regression model was used to estimate the parameters of this model.

S5 Fig represents the plot of the empirical monthly malaria cases against counts predicted by the final model by centre.

**S5 Fig: Plot of the empirical monthly malaria counts against counts predicted, by centre.** *a) centre 1=CSI AN NOUR, b) centre 2= HB TORGO, c) centre 3= IP SOBA, d) centre 4=IP TENEMANGA*
